# Acute Left Ventricular Ballooning: Tools to Differentiate Hypertrophic Cardiomyopathy with Outflow Obstruction from Neurohumoral Takotsubo Syndrome

**DOI:** 10.31083/j.rcm2405154

**Published:** 2023-05-19

**Authors:** Arushi Singh, Louai Razzouk, Daniele Massera, Mark V. Sherrid

**Affiliations:** ^1^Hypertrophic Cardiomyopathy Program, Leon H. Charney Division of Cardiology, Department of Medicine, New York University Langone Health, New York, NY 10016, USA; ^2^Interventional Cardiology, Leon H. Charney Division of Cardiology, Department of Medicine, New York University Langone Health, New York, NY 10016, USA

**Keywords:** hypertrophic cardiomyopathy, obstructive hypertrophic cardiomyopathy, takotsubo syndrome, left ventricular outflow tract obstruction, left heart catheterization

## Abstract

Despite considerable interest in the syndrome of acute left ventricular (LV) ballooning, its pathophysiology has remained ill-defined. In this review, we explore observational data describing two etiologies of acute LV ballooning: neurohumoral classic Takotsubo Syndrome (TTS), and acute severe unrelenting left ventricular outflow tract (LVOT) obstruction in patients with obstructive hypertrophic cardiomyopathy (HCM). We describe the clinical presentation and varying pathophysiology of these presentations, explore how echocardiography and cardiac catheterization may help differentiate between the two etiologies, and detail differences in management. We highlight the significant overlap as well as key differentiating features of these conditions, with the aim to improve diagnostic awareness and accuracy and appropriately tailor therapy.

## 1. Introduction

Hypertrophic cardiomyopathy (HCM) is a common genetic heart disease characterized by frequent left ventricular (LV) outflow tract obstruction [1, 2, 3]. In recent years the clinical spectrum of HCM has grown to include patients with outflow obstruction but with normal to mild LV thickness [4, 5, 6]. A subset of HCM patients, often with mild septal thickening, present acutely with apical ballooning and severe LV outflow tract (LVOT) obstruction that can progress to refractory heart failure or cardiogenic shock [7, 8]. In addition to apical ballooning, these patients share multiple clinical features with classical neurohumoral apical Takotsubo syndrome (TTS), including chest pain, electrocardiogram (ECG) changes, elevated cardiac biomarkers, and lack of angiographic coronary stenosis [9]. For this review, our guiding principle is that there are at least two proposed causes of acute apical ballooning. The first and most commonly cited hypothesis is that ballooning is caused by direct catecholamine toxicity to the cardiomyocytes, or through microvascular ischemia [10, 11]. More recently, a second hypothesis has emerged. This postulates that in some patients with underlying HCM, LV ballooning is caused by the sudden onset of latent and severe LVOT obstruction, resulting in severe afterload mismatch and supply-demand ischemia [9]. In this review, we describe how the clinical presentation of LV ballooning can obscure the difference between the two potential etiologies. The pathophysiology of each etiology will be reviewed, as well as how echocardiography and catheterization can help to define the cause of LV ballooning and thus guide the most appropriate treatment. We searched PubMed and reviewed references in published manuscripts for patients with HCM complicated by acute LV ballooning syndrome, as well as patients with TTS and suspected underlying HCM.

## 2. Case Series and Case Reports of Left Ventricular (LV) Ballooning Due to Hypertrophic Cardiomyopathy (HCM)

**Fig. 1.**
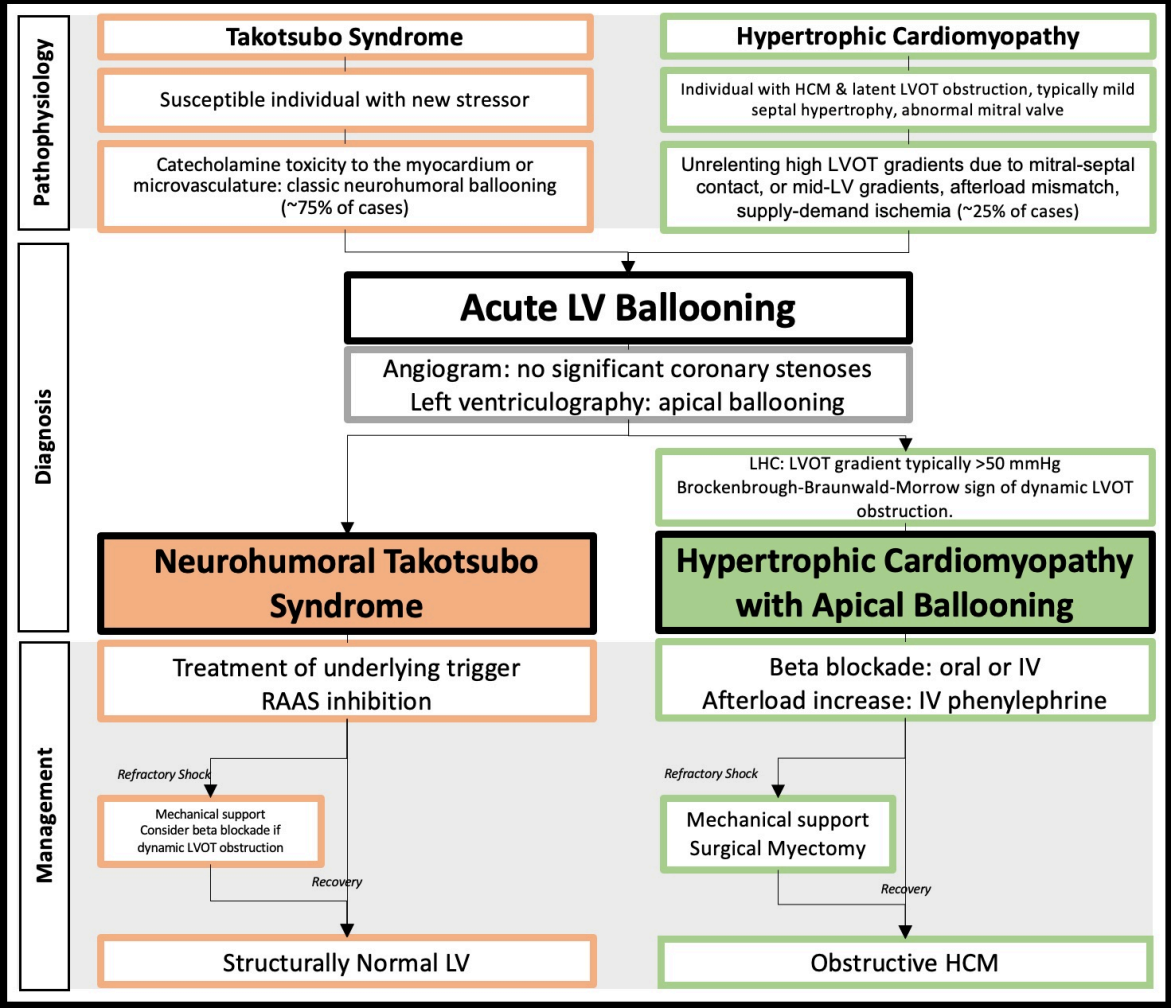
**Central figure highlighting the pathophysiological, diagnostic, and management differences between patients presenting with acute apical ballooning due to Takotsubo syndrome (TTS) versus obstructive hypertrophic cardiomyopathy (HCM)**. LVOT, left ventricular outflow tract; LV, left ventricle; LHC, left heart catheterization; IV, intravenous; RAAS, renal-angiotensin-aldosterone system.

**Fig. 2.**
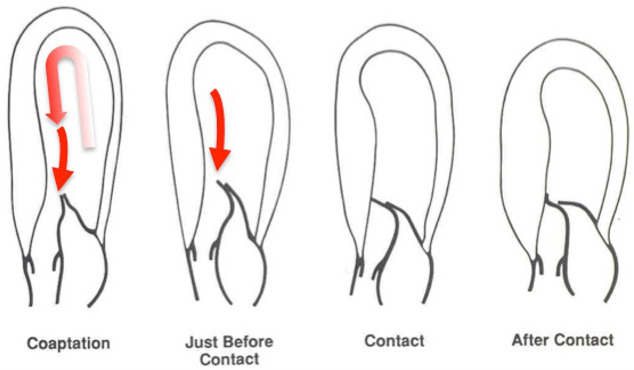
**The cardiac loop and SAM of the mitral valve**. Flow within the LV follows a U-turn redirection between the LV inflow and outflow tracts, and goes around the mitral valve. The close proximity of the LV inflow and outflow tracts makes this anatomy susceptible to small perturbations, resulting in SAM in vulnerable individuals. In obstructive HCM, the mitral valve is swept into the septum by the pushing force of flow, also known as flow drag. Flow originates from a posterior and lateral direction, redirected by the septal bulge. As it does so, flow catches the mitral valve from its ventricular aspect and sweeps it into the septum. Reproduced by permission of the authors [52]. SAM, systolic anterior motion; LV, left ventricle; HCM, hypertrophic cardiomyopathy.

Our literature search yielded case series [7, 8, 9, 12, 13, 14], case reports [15, 16, 17, 18, 19, 20, 21, 22, 23, 24, 25, 26, 27, 28, 29, 30, 31, 32, 33, 34, 35, 36, 37, 38], one prospective cohort study [39], and one systematic review [40] that described cases of obstructive HCM with LV ballooning overlapping with neurohumoral Takotsubo syndrome (TTS). We propose key features to differentiate these diagnoses, as shown in Fig. 1. Case reports describe the presence of LV outflow tract (LVOT) obstruction in TTS cases [18, 19, 21, 22, 41]. Case series suggest that up to 18% of patients with acute LV ballooning have an intraventricular pressure gradient [42, 43], with up to 25% of patients demonstrating LVOT obstruction [44]. In such cases, systolic anterior motion (SAM) of the mitral valve has been attributed to a geometric change in the shape of the LV, with a narrowed hyperkinetic outflow track and Venturi effect. However, most evidence suggests that SAM is caused by flow drag, which is the pushing force of flow that begins at low LV velocities and drives mitral leaflets into the septum, as shown in Fig. 2 [9]. Pertinent to this, a severely akinetic or severely hypokinetic LV apex generates low instantaneous ejection velocities [13]. Velocities apical to the mitral valve are not high, thus precluding a Venturi effect. Multiple case reports of LV apical ballooning in patients with HCM describe how prompt treatment of septal hypertrophy, predominantly medically but notably also after emergency myectomy or alcohol septal ablation, can lead to resolution of LVOT obstruction and rapid clinical improvement [19, 22, 23, 24]. While many of these patients were diagnosed with overlapping TTS and HCM, the almost immediate clinical improvement of shock and ballooning after surgical relief of septal hypertrophy and LVOT obstruction provides striking evidence that acute apical ballooning is in many cases a direct complication of severe obstructive HCM [8, 15, 20, 45].

## 3. Epidemiology

A major challenge in differentiating classical neurohumoral apical TTS from HCM with acute LV ballooning is the overlapping epidemiology of these syndromes. Up to 90% of TTS cases present in postmenopausal women [16]. A retrospective case series of patients who developed apical ballooning from obstructive HCM also revealed that 10 of the 13 patients (77%) with this syndrome were female, with a mean age of 64 ± 7 years [7]. A systematic review of 18 cases of overlapping TTS and HCM found that 77.8% of patients were female, with a median age of 66.2 years [40]. The overlapping demographics of patients presenting with TTS and HCM with acute LV ballooning can lead to etiologic and diagnostic misclassification. A retrospective cohort study using diagnostic codes from electronic medical records suggested that up to 30% of cases initially diagnosed as TTS were re-assessed as HCM with LV ballooning after detailed echocardiographic analysis [9]. Even with more routine presentation, obstructive HCM remains underdiagnosed in population studies, with up to one-third of cases being misclassified [46]. Consequently, a more detailed understanding of the clinical presentation, pathophysiological changes, and clinical course of HCM with apical ballooning may improve our ability to accurately diagnose obstructive HCM in this scenario.

## 4. Clinical Presentation and Echocardiography

**Fig. 3.**
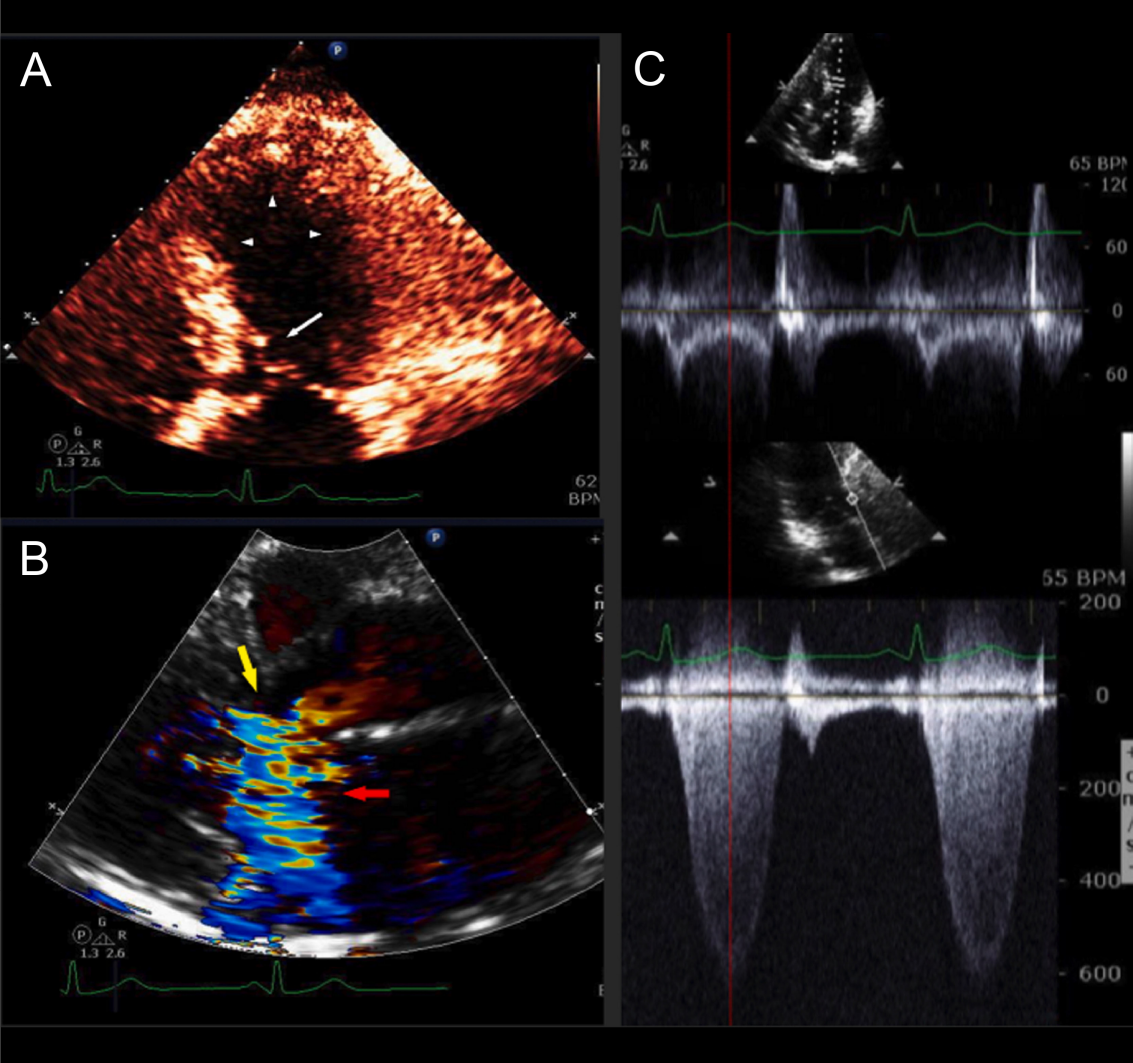
**HCM with mild asymmetric septal hypertrophy, LVOT obstruction, and acute apical ballooning syndrome**. A 63-year-old woman was admitted with near-syncope, hypotension, and NSTEMI (troponin I of 0.64 ng/mL). Coronary arteries were angiographically normal. LV cineangiography revealed acute apical ballooning. Left heart catheterization revealed an aortic pressure of 89/51 with a gradient of 100 mmHg. Following intravenous metoprolol, the patient’s outflow tract gradient improved to 40 mmHg, and her BP improved to 105/80 mmHg. A stress TTE performed 22 months after presentation demonstrated normal LV wall motion and no resting gradient. Following stress, TTE revealed mitral-septal contact and a peak LVOT gradient of 81 mmHg. This patient had very mild septal thickening HCM with acute apical ballooning due to LVOT obstruction presenting clinically as TTS. After recovery of LV systolic function, she continued to have severe LVOT gradients provocable after exercise. (A) Echocardiogram performed on admission (systolic frame) revealed apical and mid-LV ballooning with severe hypokinesis (arrowheads) and mitral-septal contact (white arrow). (B) Color Doppler (systolic frame) demonstrated severe mitral regurgitation (MR) (red arrow) and turbulence in the LV outflow tract (yellow arrow). (C) Pulsed-wave (PW; top) and continuous wave (CW; bottom) Doppler tracings obtained on admission TTE. PW Doppler at the level of the LVOT revealed a mid-systolic drop in PW Doppler velocities (“lobster claw abnormality”). The nadir in the drop of the PW Doppler corresponds to the peak of the CW gradient in the outflow tract (139 mmHg, bottom), as shown by the red dotted line. The PW velocity in mid-systole is low at 20 cm/sec, due to LV systolic dysfunction. Reproduced with permission Am J Cardiol [9]. HCM, hypertrophic cardiomyopathy; LVOT, left ventricular outflow tract; NSTEMI, Non-ST segment elevation myocardial infarction; LV, left ventricle; TTE, transthoracic echocardiogram; TTS takotsubo syndrome.

**Fig. 4.**
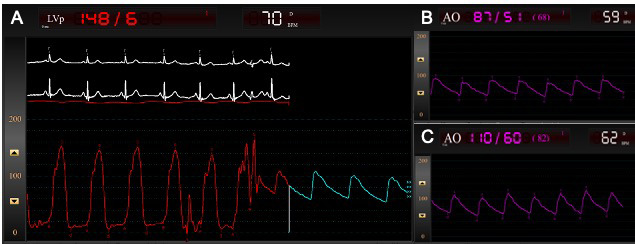
**Left heart catheterization and peak-to-peak pressure tracings of the patient described in Fig. 3, obtained using a retrograde pullback approach**. (A) Pullback method for LV and aortic pressure tracings demonstrate a 60 mmHg gradient across the LVOT. (B) Aortic pressure tracings in the catheterization laboratory demonstrate a hypotensive patient with a systemic blood pressure of 87/51 mmHg. (C) Given the concern for acute apical ballooning due to severe LVOT obstruction in HCM, the patient was administered IV metoprolol 15 mg. Fifteen minutes later, aortic pressure tracings demonstrate improved systemic blood pressures to 110/60 mmHg, as well as an improved LVOT gradient to 40 mmHg. LV, left ventricle; LVOT, left ventricular outflow tract; HCM, hypertrophic cardiomyopathy; IV, intravenous.

The clinical presentation of patients with obstructive HCM with LV ballooning share several overlapping features with TTS. Patients with these syndromes typically have chest pain, dyspnea, syncope, ST-segment changes on ECG, and elevated cardiac biomarkers, consistent with acute coronary syndrome (Fig. 3 (Ref. [9]) and Fig. 4) [7, 42]. The initial diagnostic workup for both conditions includes transthoracic echocardiogram (TTE), which typically shows apical hypokinesis with basal hyperkinesis. Coronary angiography excludes obstructive disease in both conditions [40].

Differentiating HCM with LV ballooning from TTS involves primarily the careful measurement of echocardiographic wall thickness (particularly at the basal septum), dedicated evaluation of outflow tract obstruction, echocardiographic elucidation of mitral valve pathology including SAM of mitral valve leaflets, and the evolution over time of the illness (Figs. 3,4,** Supplementary Figs. 1,2**). A potential source of diagnostic confusion is the relatively mild septal hypertrophy observed in obstructive HCM patients with apical ballooning [1, 3]. With obstructive HCM, it is important to note that septal hypertrophy, elongated mitral leaflets, papillary muscle abnormalities, and anterior displacement of the mitral leaflets persist on imaging well after the acute episode of apical ballooning. Indeed, a retrospective study noted that all 13 cases of acute apical ballooning had imaging features of obstructive HCM long after their acute presentation [7, 8, 9, 43].

## 5. Mitral Valve Anatomic Abnormalities in LV Ballooning

Typical HCM-related mitral valve abnormalities occur in approximately 30% of patients with acute LV ballooning, and specifically in patients with outflow obstruction and septal hypertrophy. This provides striking evidence that obstructive HCM is their primary underlying condition. Patients with HCM commonly have primary abnormalities of the mitral valve and apparatus [3, 20], including leaflet elongation, anomalous papillary muscle insertion, and anteriorly displaced papillary muscles [3, 20] (**Supplementary Fig. 3**). On the other hand, patients with neurohumoral TTS experience catecholamine toxicity of their cardiomyocytes and thus have no cause for mitral leaflet elongation or other primary mitral valve pathology.

## 6. Pathophysiology of LVOT Obstruction in HCM and LV Ballooning

LVOT obstruction is seen in up to 75% of patients with HCM and is associated with worse clinical outcomes [3, 47]. LVOT gradients ≥30 mmHg are indicative of obstruction and may be present at rest or with provocative maneuvers (latent obstruction) [3]. LVOT obstruction in HCM is caused by septal hypertrophy and anatomic alterations to the mitral valve apparatus, leading to an overlap of the inflow and outflow portions of the left ventricle (Fig. 2) [3, 48, 49, 50, 51, 52]. The overlap allows LV systolic flow to strike the ventricular surface of the mitral apparatus on its way to the LVOT, causing SAM and mitral-septal contact [52]. This pathophysiologic hallmark of HCM is typically diagnosed using TTE, but may also be revealed by cardiac magnetic resonance imaging (MRI), left ventriculography, and invasive hemodynamics (Figs. 3,4,** Supplementary Figs. 1,2**).

The LVOT gradient in HCM demonstrates dynamic variation, particularly in response to changes in cardiac preload, afterload, contractility, and hence catecholamines [53, 54]. In rare circumstances, patients with HCM and latent obstruction may experience severe and unrelenting increases in the LVOT gradient, progressing to LV apical ballooning and even heart failure with cardiogenic shock [7, 8, 32]. A retrospective study of two HCM treatment programs found that 0.9% of HCM patients experienced acute LV ballooning associated with dynamic LVOT obstruction and mean gradients of 92 ± 37 mmHg [7]. It is hypothesized that severe LVOT obstruction (usually >50 mmHg) independently leads to acute apical ballooning through afterload mismatch and supply-demand ischemia [15, 17]. A graphic demonstration of the corrosive effect of LV outflow obstruction by afterload mismatch is the Doppler finding of a mid-systolic drop in LV ejection velocities, the so-called “lobster claw abnormality” (Fig. 3) [55]. Myocardial ischemia is well documented in obstructive HCM and results from: (1) supply-demand ischemia from high LV pressures and decreased arterial and coronary perfusion pressures; (2) elevated LV diastolic filling pressures contributing to lower transcapillary perfusion pressures; and (3) narrowing of intramyocardial small coronary arterioles due to medial and intimal hyperplasia [56, 57, 58].

Neurohumoral TTS is believed to be mediated by high circulating catecholamine levels [10]. Patients with LV ballooning have higher levels of circulating catecholamines than those with acute myocardial infarction from occlusive coronary disease [59]. Several etiologies have been proposed to explain how excess catecholamines can mediate LV ballooning in TTS, including microvascular dysfunction, direct cardiomyocyte toxicity, paracrine influence, and myocardial inflammation [10, 59]. The current leading hypotheses are summarized in **Supplementary Table 1**. Although excess catecholamine has been implicated in the proposed mechanism for neurohumoral TTS, it is also worth noting that elevated levels can also increase the severity of LVOT obstruction, thereby leading to prolonged high gradients in patients with latent obstruction. This overlap adds to the challenge of correct diagnosis within this population.

## 7. Clinical Course and Additional Diagnostics

Although the above findings may be useful for differentiating TTS from HCM with latent obstruction and apical ballooning, the clinical evolution of the disease process over time is often the most useful for differentiating the two entities. Several case reports found that patients who presented acutely with LV apical ballooning and were diagnosed with TTS continued to show septal hypertrophy, LVOT obstruction, and mitral pathology for several weeks to months after initial presentation, and were ultimately diagnosed as HCM with obstruction [12, 25, 26, 37]. Therefore, the continued evidence of such abnormalities on imaging after acute improvement of wall motion abnormalities would indicate the presence of underlying HCM. At our center, several of these patients required late surgical myectomy and mitral repair once their symptoms proved refractory to medication.

In such cases, additional diagnostic and therapeutic modalities may improve the diagnostic accuracy. Cardiac MRI and rarely also endomyocardial biopsy have been used to diagnose underlying HCM [25, 27, 30]. Typical findings on cardiac MRI include LV hypertrophy ranging from diffuse global hypertrophy to focal segmental hypertrophy, as well as further anatomical characterization of the LVOT, papillary muscles, and subvalvular apparatus [60]. Patients with HCM may have variable papillary muscle anatomy, including anomalous anterior papillary muscle displacement and accessory papillary muscles [50, 60]. The presence of late gadolinium enhancement (LGE) by cardiac MRI in HCM can range from no LGE in patients with mild hypertrophy, to extensive LGE, thus conferring diagnostic as well as prognostic value [60]. Cardiac MRI has been used to define LV hypertrophy following acute episodes of apical ballooning. In HCM, cardiac MRI characteristically shows myocardial fibrosis and increased extracellular volumes without evidence of myocardial edema [27, 30]. Conversely, one case report described how LV apical edema mimicked apical HCM. At follow-up, cardiac MRI showed resolution of myocardial edema and wall hypertrophy, thus confirming the diagnosis of TTS [28]. This modality offers promise for distinguishing between TTS and HCM in ambiguous cases.

## 8. Diagnostic Role of Cardiac Catheterization

The current literature search found no dedicated reviews that focused on the role of diagnostic left heart catheterization in this population. A central tenet of this review is that left heart catheterization and the measurement of trans-outflow tract gradients are essential in LV ballooning and can provide etiologic diagnosis.

### 8.1 Coronary Angiography

Since the presentation of acute apical ballooning mimics an acute coronary syndrome, patients typically undergo emergency coronary angiography to evaluate for obstructive coronary artery disease (CAD) [7]. This highlights the key role of the invasive cardiologist as potentially the first physician to ascertain the cause of acute apical ballooning.

Although angiography is useful for excluding obstructive CAD, it should be noted that significant CAD (variably defined as luminal narrowing >60% or >75%) is seen in up to 24% of patients with HCM and aged >45 years, and is associated with increased mortality in HCM [61, 62, 63, 64]. In addition to evaluating CAD, coronary angiography is also useful for evaluating the septal perforator branch. Knowledge of the location and size of this branch can help to inform the discussion regarding future alcohol septal ablation versus septal myectomy for symptomatic patients with obstructive HCM [65]. The presence of concomitant CAD can alter the threshold for intervention [3], whether surgical or interventional, and angiography remains critical in guiding this decision-making [66].

### 8.2 Invasive Simultaneous Gradient Measurement

Left heart catheterization with invasive hemodynamics is recommended in patients undergoing angiography for evaluation of acute apical ballooning to assess the severity and location of outflow tract obstruction. This is measured as the difference in peak pressure between the aorta and LV (Fig. 4). The presence of an LVOT gradient at rest, with Valsalva, or after premature ventricular contractions (PVC) is suggestive of LV ballooning from obstructive HCM. Its absence on the other hand suggests neurohumoral TTS, noting however that a subset of patients with TTS may have a low level of LVOT obstruction. A caveat is that LVOT gradients are dynamic and may subside in the hours after presentation due to LV systolic dysfunction. Standard Doppler echocardiography can under-diagnose and underestimate the severity of LV obstruction in patients with mid-LV obstructive HCM due to signal void from the absence or diminution of flow across the mid-left ventricle [13, 39]. In these instances, invasive hemodynamic measurements may detect the presence of high invasive catheterization gradients in the absence of high Doppler velocities [13, 39]. Alternatively, patients with a hypercontractile LV may generate high velocities on Doppler echocardiography due to complete LV emptying in the absence of true LV cavitary obstruction. This can be elucidated as the absence of a pressure gradient on cardiac catheterization [38].

Optimal hemodynamic assessment of LVOT obstruction is contingent upon using the proper equipment to obtain a high-quality hemodynamic assessment and to minimize error. All catheters should be intermittently flushed and rebalanced to the zero baseline. Interventional cardiologists should take care to monitor pressure tracings for signs of dampened or unusual pressure contours which can occur due to the catheter position or to entrapment [67]. In general, catheters with distal side holes are favored over catheters with end-holes (i.e., coronary artery catheters) because they minimize damping artifact [67].

The dual lumen Langston catheter had been used to simultaneously measure invasive LV and aortic pressures [14, 68]. It consisted of a 5F outer pigtail catheter on a 4F inner catheter with two hubs at each of the proximal and distal ports to perform simultaneous invasive pressure measurements [69]. However, the Langston catheter was recalled in 2020 due to safety concerns regarding the risk of potential inner catheter separation [70]. Following this recall, the invasive measurement of pressure gradients now consists of a variety of techniques, as detailed below.

Our preferred method for quantifying the LVOT pressure gradient involves the use of a 125 cm 4F pigtail catheter in the LV through a 6F guiding catheter in the aorta. This is commonly referred to as the “mother-child” technique. The 4F pigtail may be replaced with a 135 cm 0.35 support catheter such as a Quickcross or Trailblazer in the LV, or with a 125 cm 4F MPA catheter in the LV apex in the case of patients with suspected mid-cavitary obstruction. This technique uses a single arterial access site and commonly available traditional catheters, is inexpensive, and demonstrates high fidelity when compared to pressure gradients measured by a dual lumen catheter [71]. A 6F guiding catheter in the aorta can also be paired with an fractional flow reserve, instantaneous wave-free ratio, diastolic hyperemia-free ratio, or relative full-cycle ratio (FFR, iFR, DFR, or RFR) wires in the LV for simultaneous pressure recordings [14, 72, 73].

A 6F Swan-Ganz catheter may be advanced over a Runthrough wire via a 5F or 6F arterial sheath and positioned with its proximal port (typically situated in the right atrium) in the aorta and its distal port (typically situated in the pulmonary artery) in the LV. Once positioned in the LV, the Swan-Ganz catheter balloon may be inflated in order to move the catheter away from the LV wall and minimize the damping artifact [14, 72].

Peak-to-peak gradients can be obtained in the retrograde pullback approach when simultaneous recordings cannot be obtained at the level of the LV and aorta. These are assessed as the difference between peak LV systolic pressure and peak central aortic pressure (Fig. 4) [74]. Alternatively, interventionalists may engage a 6F to 8F pigtail catheter via the femoral artery, with pressures equalized between the ascending aorta and femoral sheath side-arm. Intracavitary gradient measurements can be obtained by advancing the pigtail catheter to the LV apex, followed by careful pullback of the catheter from the apex to the ascending aorta to quantify peak gradients [39]. Studies have demonstrated a high fidelity and close correlation of peak-to-peak catheterization measurements of LVOT gradient using dual catheter techniques to peak instantaneous echocardiographic gradient measurements [74].

Additional maneuvers include obtaining access at two arterial sites and advancing two separate catheters into the aorta and LV to obtain simultaneous pressure recordings. However, this technique poses an increased risk of access site complications [14]. We caution that obtaining simultaneous LV and peripheral access site measurements (directly via radial or femoral sheaths) is less accurate than the above techniques due to the presence of peripheral amplification phenomenon and occasionally peripheral arterial disease [67].

### 8.3 The Brockenbrough-Braunwald-Morrow Sign

The Brockenbrough-Braunwald-Morrow (BBM) sign was first described in 1961. It can be elicited using invasive hemodynamics and is now well established as a hemodynamic feature of HCM [75]. This sign is characterized by an increased LVOT gradient in a post-PVC beat, together with an increased LV pressure and a concurrent failure to increase, or a decrease in the arterial pulse pressure [3, 75]. Post-extrasystolic potentiation following a PVC results in increased LV contractility, which then exacerbates outflow tract obstruction in patients with HCM [76]. The BBM sign is therefore particularly useful for defining the subset of HCM patients with dynamic LVOT obstruction. It can also be used to guide and monitor the therapeutic response to alcohol septal ablation and surgical myectomy [77, 78]. Although the provocative maneuver of isoproterenol infusion to elicit dynamic obstruction is sometimes performed during catheterization of patients with obstructive HCM [74], this should not be performed in acute apical ballooning. There is currently no data regarding the sensitivity and specificity of the BBM maneuver for HCM. Case reports have suggested the BBM sign confers additive utility in differentiating HCM with apical ballooning from TTS [24, 29]. However, isolated case reports have described this sign in patients following mitral valve repair [79], as well as in a patient with TTS and dynamic LVOT obstruction [80]. Further studies are therefore required to determine the specificity of the BBM sign for differentiating HCM from other forms of dynamic obstruction, including TTS.

## 9. Management

The ability to differentiate LV apical ballooning in obstructive HCM versus neurohumoral TTS is particularly important for guiding appropriate treatment.

### 9.1 Conservative Management of Apical Ballooning from LVOT Obstruction

The initial treatment of patients with HCM and acute apical ballooning is focused on identifying and reversing any triggers, including fluid resuscitation for hypovolemia and rhythm control for new arrhythmias [8]. Beta blockers form the mainstay of therapy in clinically stable cases [3]. In a previously published case series by our group, IV beta blockade (administered in the form of IV metoprolol or esmolol infusion) was found to be useful for reversing acute apical ballooning, even in HCM patients with borderline blood pressures [8] (Fig. 4). For patients with hypotension or shock, we recommend vasoconstrictive vasopressors such as phenylephrine for hemodynamic support, together with concomitant beta blockade for negative inotropy [8]. Vasopressors with inotropic properties and inotropic agents are contraindicated in these patients due to the risk of exacerbating LVOT obstruction [9].

### 9.2 Mechanical Support in Apical Ballooning due to LVOT Obstruction

Patients who progress to cardiogenic shock may benefit from mechanical circulatory support and emergency surgery to relieve LVOT obstruction, as opposed to performing septal ablation [8, 9]. Specific forms of mechanical circulatory support have not been studied in this population, although a case series described patients who were placed on intra-aortic balloon pumps or venoarterial extra corporeal membrane oxygenation (VA-ECMO) for cardiogenic shock due to acute apical ballooning [8]. Intra-aortic balloon pump counter-pulsation, by decreasing afterload, and Impella support, by decreasing preload, may exacerbate LVOT obstruction [8]. Among the available mechanical circulatory support devices, VA-ECMO may be the favored option as it can offer full circulatory support while mildly increasing afterload, which could therefore benefit patients with obstructive HCM physiology [15]. Moreover, VA-ECMO can act as a bridge to patient recovery or to septal reduction therapy [8, 15]. These management practices are guided by observational studies and case series, and have been extrapolated from the current knowledge base in patients with HCM without acute apical ballooning.

### 9.3 Septal Reduction Therapy for Acute Apical Ballooning in LVOT Obstruction

If patients cannot be weaned from mechanical support, they should be considered for urgent septal reduction therapy with surgical myectomy, with consideration for possible ancillary mitral valve shortening. It should be noted that patients who present with pathophysiology consistent with obstructive HCM with acute apical ballooning typically respond immediately to septal reduction therapy. Such patients show rapid improvement in obstructive gradients, LV dysfunction and shock, in contrast to TTS patients with shock who typically recover over a subacute time period [19, 24]. As described above, interventional cardiologists play a critical role in defining the anatomy and therapeutic targets for septal reduction therapy.

### 9.4 Conservative Management of Neurohumoral TTS

Large, observational registry data do not support the routine use of beta blockade for improving the survival of patients with TTS [81]. However, there are no randomized controlled trials of beta blockade in these patients [9]. Observational studies on a total of 43 patients found that beta blocker infusion was associated with decreased obstructive gradients and improved ejection fraction in TTS patients with outflow obstruction [82, 83]. These studies suggest a potential role for beta blockade in this subset of patients with neurohumoral TTS, however this observation remains preliminary and observational [82]. Non-randomized, observational registry data on 1750 patients with apical ballooning have revealed that RAAS inhibitors are associated with improved patient survival after 1 year, although this has yet to be evaluated in randomized studies. The renal-angiotensin-aldosterone system (RAAS) inhibitors were presumably not given to patients with LVOT obstruction [81].

### 9.5 Shock in Neurohumoral TTS

Given that the pathophysiological basis of neurohumoral TTS is catecholamine excess, catecholaminergic inotropes are not favored for the management of shock in TTS and have been associated with increased mortality in registry data [84]. Levosimendan, a non-catecholaminergic inotropic agent that potentiates the effect of calcium in the sarcomere, could be considered for the treatment of shock, although it is currently not approved for use in North America. Mechanical support is indicated if the shock persists. There is no generally reported preference for the type of mechanical circulatory support for TTS patients, and hence the severity of shock and local expertise should be used to guide procedural considerations.

## 10. Conclusions

As highlighted in this review, there may be significant overlap in the clinical presentation of neurohumoral TTS and obstructive HCM with apical ballooning. Key features that may help to differentiate these entities include the presence of LVOT obstruction, mitral valve pathology, and the persistence of echocardiographic HCM following acute presentation. Since up to 30% of affected patients may be misdiagnosed, it is important for invasive cardiologists and echocardiographers to explore the key distinguishing features of obstructive HCM from neurohumoral TTS to better determine the diagnosis and tailor therapy. Patients with HCM and LVOT obstruction are treated with intravenous beta blockade and phenylephrine for hypotension. In contrast, patients with neurohumoral TTS are given supportive treatment in the acute setting. Observational data supports the role of RAAS inhibitors in this population, presumably after excluding patients with LVOT obstruction. In patients with HCM and apical ballooning complicated by shock, mechanical support that decreases afterload or preload should be avoided, and VA-ECMO may be preferred if available. Refractory shock may improve after septal reduction therapy, which is often performed with ancillary mitral shortening. Treatment of such patients and their unique pathology requires familiarity with the obstructive HCM ballooning syndrome, as well as a trained and equipped team that is ready to intervene.

## Supplementary Material

Supplementary material associated with this article can be found, in the online version, at https://doi.org/10.31083/j.rcm2405154.
