# Supplementary material for: Acute Left Ventricular Ballooning: Tools to Differentiate Hypertrophic Cardiomyopathy with Outflow Obstruction from Neurohumoral Takotsubo Syndrome
Source: Rev Cardiovasc Med. 2023 May 19;24(5):154. doi: 10.31083/j.rcm2405154 (PMC11273027; doi:10.31083/j.rcm2405154)
Supplement: Supplementary file 1 [file 2153-8174-24-5-154-s1.zip › 2153-8174-24-5-154-s1.docx]

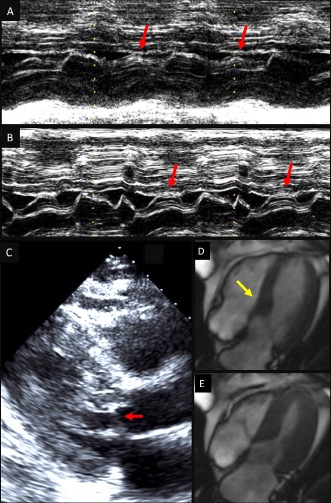


**Supplementary Fig. 1. A 58-year-old man with HCM and mild resting outflow tract gradients and severe, provocable outflow tract gradients, developed severe persistent resting outflow tract obstruction, acute apical LV ballooning, and cardiogenic shock**. The patient was treated with β-blockade and verapamil, with a subsequently stable course. (A) An M-mode tracing at the level of the mitral valve leaflets was obtained prior to the patient’s acute presentation, demonstrating systolic anterior motion (SAM) of the mitral valve with transient mitral-septal contact (red arrows). (B) An M-mode tracing at the level of the mitral valve leaflets was performed prior to the acute presentation after Valsalva maneuver, showing prolonged mitral-septal contact. The patient’s resting LV outflow gradient was 40 mmHg, and increased to 84 mmHg after Valsalva maneuver. (C) A systolic parasternal long-axis view obtained after Valsalva maneuver demonstrates SAM and mitral-septal contact (red arrow). (D) Cardiac MRI in the diastolic 4-chamber view reveals a 17-mm anterior septal bulge (yellow arrow). (E) Cardiac MRI in the systolic frame demonstrates normal LV systolic function. Reproduced with permission American Heart Association [8].


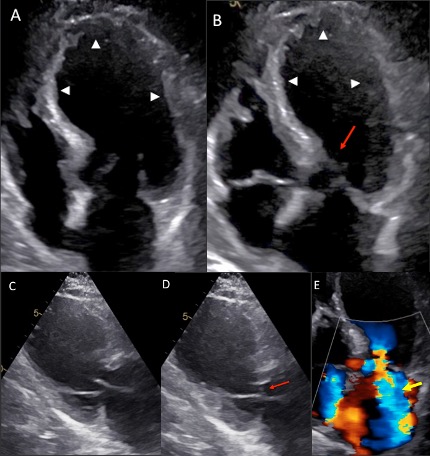


**Supplementary Fig. 2. Echocardiographic images from the same patient as Supplementary Fig. 1, two years after initial presentation.** Following 3 hours of physical labor, the patient developed resting chest discomfort, hypotension (78/50 mmHg), pulmonary edema, new anterior ST-segment elevations with troponin I of 6 ng/mL, and cardiogenic shock. He was treated with intravenous (IV) metoprolol and IV phenylephrine for 2 days, and ultimately discharged on oral β-blockade therapy. A TTE performed 6 weeks later demonstrated HCM with mild septal hypertrophy (13–14 mm), normal LV systolic function, resting SAM, and LVOT gradient of 40 mm Hg. Over the ensuing months, he had limiting symptoms and higher resting gradients with mitral regurgitation despite pharmacologic treatment, and was referred for extended surgical septal myectomy, with improvement of symptoms. He is now New York Heart Association class II, 4 months after surgery. (A) Diastolic apical 4-chamber view revealing acute apical hypokinesis & ballooning and mid-LV hypokinesis (arrowheads). There is mild asymmetric septal hypertrophy with an elongated anterior mitral valve leaflet (29 mm). (B) Systolic apical 4-chamber view showing dyskinetic apical and akinetic mid-LV segments (arrowheads), SAM of the mitral valve, mitral-septal contact (red arrow), and a resting LVOT gradient of 135 mmHg. (C) Diastolic parasternal long-axis view showing LV apical and mid segmental ballooning. (D) Systolic parasternal long-axis view showing LV apical and mid segmental ballooning and mitral-septal contact (red arrow). (E) Severe, laterally directed mitral regurgitation (yellow arrow). Reproduced with permission American Heart Association [8].

**
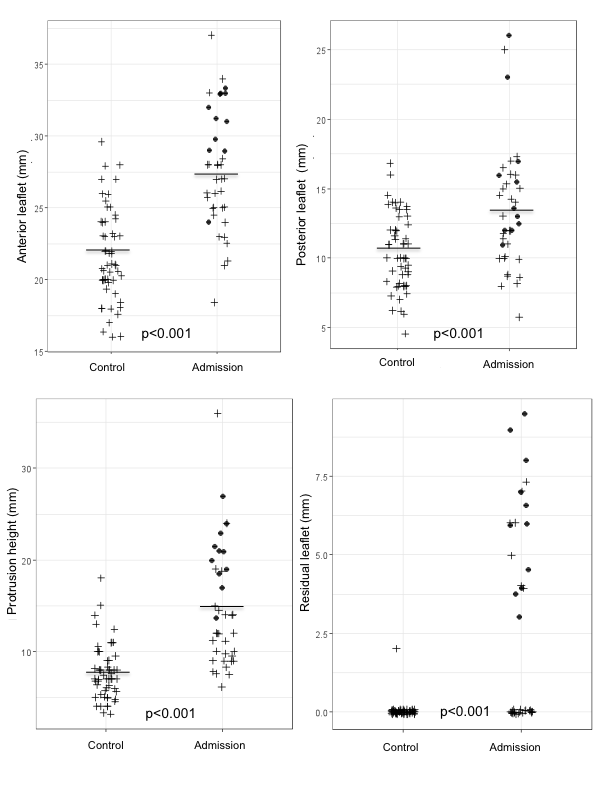
**

**Supplementary Fig. 3. Scatter plots of anterior mitral leaflet length, posterior mitral leaflet length, protrusion height, and residual leaflet length in 60 control and 44 Takotsubo patients who presented with acute apical ballooning.** Patients with SAM of the mitral valve are shown with solid black circles. Protrusion height is defined as distance from mitral annulus to tip of longest mitral leaflet. Residual leaflet length is defined as the length of the portion of the mitral valve anterior leaflet that extends past the coaptation point. Reproduced with permission from Am J Cardiol [9].

Supplementary Table 1. Proposed Pathophysiologic Mechanisms for Neurohumoral Takotsubo Syndrome.

| **Proposed Mechanism** | **Details** | **References** |
| --- | --- | --- |
| Microvascular Dysfunction | Observational studies describe an association between TTS and microvascular perfusion abnormalities as evaluated by myocardial perfusion imaging, contrast echocardiography, coronary flow reserve & microvascular testing. Unclear whether microvascular dysfunction is primary pathological cause of TTS or if it occurs secondary to myocardial dysfunction & inflammation. | 1-11 |
| Epicardial Coronary Spasm | Observed in rare case reports, though not in the majority of patients with TTS. Felt unlikely to be major pathophysiological mechanism as the distribution of wall motion abnormalities in TTS do not correlate to epicardial territories. | 2, 3, 12 |
| Direct Cardiomyocyte Toxicity | Increased catecholamine-induced ß-adrenergic receptor signaling, believed to be present in higher density at apical compared to basal segments. Increased ß receptor signaling leads to molecular switching & activation of the G_i_ pathway, which exerts a negative inotropic effect. | 1, 3, 13, 14 |
| Paracrine Influence | Acutely elevated LV intracavitary pressures lead to endocardial injury. Endocardial damage leads to paracrine factor release which mediates dysfunction across adjacent myocardial tissue. | 1, 15, 16 |
| Myocardial Inflammation | - Myocardial macrophage inflammatory infiltrate with M1 (inflammatory) macrophages and increase in systemic pro-inflammatory cytokines, which may persist for at least 5 months  - Cases of immune checkpoint inhibitor use triggering TTS suggest a causative role of pro-inflammatory pathways | 1, 3, 17-21 |

***Supplementary References***

1. Omerovic E, Citro R, Bossone E, Redfors B, Backs J, Bruns B, Ciccarelli M, Couch LS, Dawson D, Grassi G, Iacoviello M, Parodi G, Schneider B, Templin C, Ghadri JR, Thum T, Chioncel O, Tocchetti CG, van der Velden J, Heymans S, Lyon AR. Pathophysiology of Takotsubo syndrome - a joint scientific statement from the Heart Failure Association Takotsubo Syndrome Study Group and Myocardial Function Working Group of the European Society of Cardiology - Part 1: overview and the central role for catecholamines and sympathetic nervous system. Eur J Heart Fail. 2022 Feb;24(2):257-273. doi: 10.1002/ejhf.2400. Epub 2022 Feb 16. PMID: 34907620.

2. Omerovic E, Citro R, Bossone E, Redfors B, Backs J, Bruns B, Ciccarelli M, Couch LS, Dawson D, Grassi G, Iacoviello M, Parodi G, Schneider B, Templin C, Ghadri JR, Thum T, Chioncel O, Tocchetti CG, van der Velden J, Heymans S, Lyon AR. Pathophysiology of Takotsubo syndrome - a joint scientific statement from the Heart Failure Association Takotsubo Syndrome Study Group and Myocardial Function Working Group of the European Society of Cardiology - Part 2: vascular pathophysiology, gender and sex hormones, genetics, chronic cardiovascular problems and clinical implications. Eur J Heart Fail. 2022 Feb;24(2):274-286. doi: 10.1002/ejhf.2368. Epub 2021 Nov 3. PMID: 34655287.

3. Lyon AR, Citro R, Schneider B, Morel O, Ghadri JR, Templin C, Omerovic E. Pathophysiology of Takotsubo Syndrome: JACC State-of-the-Art Review. J Am Coll Cardiol. 2021 Feb 23;77(7):902-921. doi: 10.1016/j.jacc.2020.10.060. PMID: 33602474.

4. Dote K, Sato H, Tateishi H, Uchida T, Ishihara M. [Myocardial stunning due to simultaneous multivessel coronary spasms: a review of 5 cases]. J Cardiol. 1991;21(2):203-14. Japanese. PMID: 1841907.

5. Ito K, Sugihara H, Kinoshita N, Azuma A, Matsubara H. Assessment of Takotsubo cardiomyopathy (transient left ventricular apical ballooning) using 99mTc-tetrofosmin, 123I-BMIPP, 123I-MIBG and 99mTc-PYP myocardial SPECT. Ann Nucl Med. 2005 Sep;19(6):435-45. doi: 10.1007/BF02985570. PMID: 16248379.

6. Galiuto L, De Caterina AR, Porfidia A, Paraggio L, Barchetta S, Locorotondo G, Rebuzzi AG, Crea F. Reversible coronary microvascular dysfunction: a common pathogenetic mechanism in Apical Ballooning or Tako-Tsubo Syndrome. Eur Heart J. 2010 Jun;31(11):1319-27. doi: 10.1093/eurheartj/ehq039. Epub 2010 Mar 9. PMID: 20215125.

7. Martin EA, Prasad A, Rihal CS, Lerman LO, Lerman A. Endothelial function and vascular response to mental stress are impaired in patients with apical ballooning syndrome. J Am Coll Cardiol. 2010 Nov 23;56(22):1840-6. doi: 10.1016/j.jacc.2010.03.107. PMID: 21087714; PMCID: PMC3786427.

8. Ghadri JR, Dougoud S, Maier W, Kaufmann PA, Gaemperli O, Prasad A, Lüscher TF, Templin C. A PET/CT-follow-up imaging study to differentiate takotsubo cardiomyopathy from acute myocardial infarction. Int J Cardiovasc Imaging. 2014 Jan;30(1):207-9. doi: 10.1007/s10554-013-0311-x. Epub 2013 Oct 22. PMID: 24146288.

9. Patel SM, Lerman A, Lennon RJ, Prasad A. Impaired coronary microvascular reactivity in women with apical ballooning syndrome (Takotsubo/stress cardiomyopathy). Eur Heart J Acute Cardiovasc Care. 2013 Jun;2(2):147-52. doi: 10.1177/2048872613475891. PMID: 24222824; PMCID: PMC3821803.

10. Uchida Y, Egami H, Uchida Y, Sakurai T, Kanai M, Shirai S, Nakagawa O, Oshima T. Possible participation of endothelial cell apoptosis of coronary microvessels in the genesis of Takotsubo cardiomyopathy. Clin Cardiol. 2010 Jun;33(6):371-7. doi: 10.1002/clc.20777. PMID: 20556810; PMCID: PMC6653365.

11. Jaguszewski M, Osipova J, Ghadri JR, Napp LC, Widera C, Franke J, Fijalkowski M, Nowak R, Fijalkowska M, Volkmann I, Katus HA, Wollert KC, Bauersachs J, Erne P, Lüscher TF, Thum T, Templin C. A signature of circulating microRNAs differentiates takotsubo cardiomyopathy from acute myocardial infarction. Eur Heart J. 2014 Apr;35(15):999-1006. doi: 10.1093/eurheartj/eht392. Epub 2013 Sep 17. PMID: 24046434; PMCID: PMC3985061.

12. Fiol M, Carrillo A, Rodriguez A, Herrero J, García-Niebla J. Left ventricular ballooning syndrome due to vasospasm of the middle portion of the left anterior descending coronary artery. Cardiol J. 2012;19(3):314-6. doi: 10.5603/cj.2012.0055. PMID: 22641552.

13. Daaka Y, Luttrell LM, Lefkowitz RJ. Switching of the coupling of the beta2-adrenergic receptor to different G proteins by protein kinase A. Nature. 1997 Nov 6;390(6655):88-91. doi: 10.1038/36362. PMID: 9363896.

14. Redfors B, Shao Y, Ali A, Omerovic E. Current hypotheses regarding the pathophysiology behind the takotsubo syndrome. Int J Cardiol. 2014 Dec 20;177(3):771-9. doi: 10.1016/j.ijcard.2014.10.156. Epub 2014 Oct 25. PMID: 25466564.

15. Brutsaert DL. Tako-Tsubo cardiomyopathy. Eur J Heart Fail. 2007 Aug;9(8):854; author reply 855. doi: 10.1016/j.ejheart.2007.06.008. Epub 2007 Jul 24. PMID: 17652022.

16. Brutsaert DL. Cardiac endothelial-myocardial signaling: its role in cardiac growth, contractile performance, and rhythmicity. Physiol Rev. 2003 Jan;83(1):59-115. doi: 10.1152/physrev.00017.2002. PMID: 12506127.

17. Scally C, Abbas H, Ahearn T, Srinivasan J, Mezincescu A, Rudd A, Spath N, Yucel-Finn A, Yuecel R, Oldroyd K, Dospinescu C, Horgan G, Broadhurst P, Henning A, Newby DE, Semple S, Wilson HM, Dawson DK. Myocardial and Systemic Inflammation in Acute Stress-Induced (Takotsubo) Cardiomyopathy. Circulation. 2019 Mar 26;139(13):1581-1592. doi: 10.1161/CIRCULATIONAHA.118.037975. PMID: 30586731; PMCID: PMC6438459.

18. Ederhy S, Dolladille C, Thuny F, Alexandre J, Cohen A. Takotsubo syndrome in patients with cancer treated with immune checkpoint inhibitors: a new adverse cardiac complication. Eur J Heart Fail. 2019 Jul;21(7):945-947. doi: 10.1002/ejhf.1497. PMID: 31353808.

19. Neil C, Nguyen TH, Kucia A, Crouch B, Sverdlov A, Chirkov Y, Mahadavan G, Selvanayagam J, Dawson D, Beltrame J, Zeitz C, Unger S, Redpath T, Frenneaux M, Horowitz J. Slowly resolving global myocardial inflammation/oedema in Tako-Tsubo cardiomyopathy: evidence from T2-weighted cardiac MRI. Heart. 2012 Sep;98(17):1278-84. doi: 10.1136/heartjnl-2011-301481. Epub 2012 Jul 11. PMID: 22791656.

20. Wilson HM, Cheyne L, Brown PAJ, Kerr K, Hannah A, Srinivasan J, Duniak N, Horgan G, Dawson DK. Characterization of the Myocardial Inflammatory Response in Acute Stress-Induced (Takotsubo) Cardiomyopathy. JACC Basic Transl Sci. 2018 Dec 31;3(6):766-778. doi: 10.1016/j.jacbts.2018.08.006. PMID: 30623136; PMCID: PMC6314973.

21. Schoemaker IE, Meulemans AL, Andries LJ, Brutsaert DL. Role of endocardial endothelium in positive inotropic action of vasopressin. Am J Physiol. 1990 Oct;259(4 Pt 2):H1148-51. doi: 10.1152/ajpheart.1990.259.4.H1148. PMID: 2221123.
